# Structural and biochemical analysis of atypically low dephosphorylating activity of human dual-specificity phosphatase 28

**DOI:** 10.1371/journal.pone.0187701

**Published:** 2017-11-09

**Authors:** Bonsu Ku, Won Hong, Chae Won Keum, Myeongbin Kim, Hyunyeol Ryu, Donghwan Jeon, Ho-Chul Shin, Jae Hoon Kim, Seung Jun Kim, Seong Eon Ryu

**Affiliations:** 1 Disease Target Structure Research Center, Korea Research Institute of Bioscience and Biotechnology, Daejeon, Republic of Korea; 2 Department of Bioscience, University of Science and Technology KRIBB School, Daejeon, Republic of Korea; 3 Department of Bioengineering, College of Engineering, Hanyang University, Seoul, Republic of Korea; 4 Department of Biotechnology, College of Applied Life Science, SARI, Jeju National University, Jeju-do, Republic of Korea; University of Washington, UNITED STATES

## Abstract

Dual-specificity phosphatases (DUSPs) constitute a subfamily of protein tyrosine phosphatases, and are intimately involved in the regulation of diverse parameters of cellular signaling and essential biological processes. DUSP28 is one of the DUSP subfamily members that is known to be implicated in the progression of hepatocellular and pancreatic cancers, and its biological functions and enzymatic characteristics are mostly unknown. Herein, we present the crystal structure of human DUSP28 determined to 2.1 Å resolution. DUSP28 adopts a typical DUSP fold, which is composed of a central β-sheet covered by α-helices on both sides and contains a well-ordered activation loop, as do other enzymatically active DUSP proteins. The catalytic pocket of DUSP28, however, appears hardly accessible to a substrate because of the presence of nonconserved bulky residues in the protein tyrosine phosphatase signature motif. Accordingly, DUSP28 showed an atypically low phosphatase activity in the biochemical assay, which was remarkably improved by mutations of two nonconserved residues in the activation loop. Overall, this work reports the structural and biochemical basis for understanding a putative oncological therapeutic target, DUSP28, and also provides a unique mechanism for the regulation of enzymatic activity in the DUSP subfamily proteins.

## Introduction

Protein tyrosine phosphorylation is one of the common and critical post-translational modifications that is controlled by coordination of members of two large protein families, protein tyrosine kinases (PTKs) and protein tyrosine phosphatases (PTPs) [1]. Thus far, more than 100 PTPs have been reported to be encoded in the human genome [2]. A large proportion of these PTPs has been shown to dephosphorylate not only phosphorylated tyrosine but also other substrates such as phosphorylated serine and threonine or phosphoinositides [1,2]. Dual-specificity phosphatases (DUSPs), which are known to remove a phosphate group from both phosphotyrosine and phosphoserine/threonine, constitute a large subfamily of PTPs [3]. DUSP proteins participate in various areas of regulation of intracellular signaling and other physiological processes that are associated with cell growth, differentiation, apoptosis, and transformation, by modulating protein-protein interactions, protein stability and localization, and enzymatic activity [3]. It is also known that deficiencies in DUSP functions are implicated in the development and progression of diverse diseases, including diabetes, atherosclerosis, inflammatory disorders, and especially a wide variety of cancers [4,5].

DUSP proteins commonly share considerable structural homology in their catalytic domain, which has a characteristic overall protein fold, contains a conserved phosphate-binding loop (P-loop) with the PTP signature motif HCxxGxxR, and is featured by a shallow and broad active site pocket for the accommodation of the three types of phosphorylated amino acids [6]. Nonetheless, structural studies have also revealed that differences in structural details among the catalytic domains such as the conformation of the active site region [6–9] and the shape, size, and depth of the active site pocket [10–13] affect the magnitude of the enzymatic activity and substrate preference, which should determine the proteins’ functions in cells. DUSP1−2, DUSP4−10, and DUSP16 are classified as mitogen-activated protein kinase (MAPK) phosphatases (MKPs), which commonly contain an N-terminal MAPK binding domain (MKB) that is crucial for modulating their enzymatic activity and functions in the MAPK signaling cascade [2,6,14]. On the other hand, DUSP3, DUSP11−15, DUSP18−19, DUSP21−23b, and DUSP26−28 are categorized as atypical DUSPs that have neither MKB nor any other regulatory domain [2,6], with the exception of DUSP12, which contains a C-terminal FYVE domain [15].

DUSP28, also known as VHP, is a member of the subfamily of atypical DUSPs and contains a single catalytic domain, whose precise biological functions and enzymatic characteristics are largely unknown. Recent reports indicated that DUSP28 is implicated in hepatocellular carcinoma progression [16] and in migratory activity and drug resistance of pancreatic cancer cells [17,18], suggesting that this protein may be deeply involved in oncogenesis and could be a putative therapeutic target in some cancers. Although the crystal structure of mouse DUSP28 has been determined and deposited in the Protein Data Bank (PDB) as a result of a structural genomics study on protein phosphatases [19], neither structural nor biochemical analysis of DUSP28 accompanied that report. Herein, we present the crystal structure of human DUSP28 determined to 2.1 Å resolution; these data provided the basis for our in-depth atomic structural and biochemical analyses of this protein. We found that despite the similarity of the overall fold and the active loop conformation to those of canonical DUSP proteins, DUSP28 has an exceptionally low phosphatase activity. It can be accounted for by the low accessibility of the active site pocket of DUSP28. This problem may be caused by the substrate accommodation hindrance by the presence of nonconserved bulky residues within the PTP signature motif, which differentiates DUSP28 from other DUSP proteins.

## Results

### Crystal structure of human DUSP28

This human DUSP28 structure containing two mutations (R59Q and C103S; see Materials and Methods) was determined to 2.1 Å resolution (Table 1). An asymmetric unit of the crystal contains two molecules of DUSP28, which are designated as molecules A and B in S1A Fig. Among all the 176 residues of human DUSP28, residues 12−159 of molecule A and residues 19−161 of molecule B are visible in the structure. At a glance, molecules A and B appear to interact with each other, thus forming a dimeric structure (S1A Fig, top). Nevertheless, analysis of the putative dimeric interface using the Protein Interfaces, Surfaces, and Assemblies (PISA) server [20] showed that its complexation significance score is 0 (S1A Fig, bottom), suggesting that this dimer is a crystallographic artifact rather than a physiological complex. To experimentally verify the possibility of DUSP28 homodimerization in solution, DUSP28 was first subjected to the acidic native gel electrophoresis. Only a single discrete protein band was shown on the gel stained with Coomassie Blue (S1B Fig). We next carried out size exclusion chromatography-multiangle light scattering (SEC-MALS) analysis using protein samples diluted to a three different concentration (2, 10, and 20 mg/mL). Only a single protein peak with 100% mass fraction was observed in each experiment, whose molecular weight was calculated as the monomeric form (S1C Fig). Collectively, the observed dimeric structure of DUSP28 appears to be a non-physiological crystallographic artifact, and it is presumed that DUSP28 exists as a monomeric form in solution. Because molecules A and B overlap each other fairly well when superposed with a root mean square deviation (RMSD) of 0.44 Å over 141 aligned residues, we used the crystal structure of molecule A as a template for the structural analysis of human DUSP28 described below.

**Table 1:** Data collection and structure refinement statistics.

Data Collection	DUSP28	DUSP28(Y102H)
**Space group**	*P*3_1_21	*P*3_1_21
**Unit cell dimensions**		
**a, b, c (Å)**	78.95, 78.95, 90.26	79.36, 79.36, 90.50
**α, β, γ (**^**o**^**)**	90, 90, 120	90, 90, 120
**Resolution (Å)**	50.0−2.1 (2.14−2.10)^b^	50.0−2.4 (2.44−2.40)^b^
***R***_**sym**_^**a**^ **(%)**	5.1 (28.8)	6.3 (30.9)
***I*/σ(*I*)**	47.2 (4.4)	22.2 (3.2)
**Completeness (%)**	99.3 (99.1)	93.5 (89.4)
**Redundancy**	8.0	3.9
**Refinement**		
**Resolution (Å)**	50.0−2.1	50.0−2.4
**Number of reflections**	19301	12461
***R***_**work**_^**c**^**/*R***_**free**_ **(%)**	21.9/26.9	18.7/24.0
**Number of atoms**		
**Protein**	2153	2174
**Water and ions**	52	60
**R.m.s deviations**		
**Bond lengths (Å)**	0.010	0.009
**Bond angles (**^**o**^**)**	1.361	1.033
**Ramachandran plot (%)**		
**Most favored region**	95.5	94.1
**Additionally allowed region**	4.5	5.9
**Average B-values (Å**^**2**^**)**		
**Protein**	50.6	44.3
**Water and ions**	47.9	47.7

^a^*R*_sym_ = Σ |*I*_obs_—*I*_avg_| / *I*_obs_, where *I*_obs_ is the observed intensity of individual reflection and *I*_avg_ is the average across symmetry equivalents.

^b^The numbers in parentheses are statistics from the shell with the highest resolution.

^c^*R*_work_ = Σ ||*F*_o_|—|*F*_c_|| / Σ |*F*_o_|, where |*F*_o_| and |*F*_c_| are the observed and calculated structure factor amplitudes, respectively. *R*_free_ was calculated with 10% of the data.

**Fig 1:**
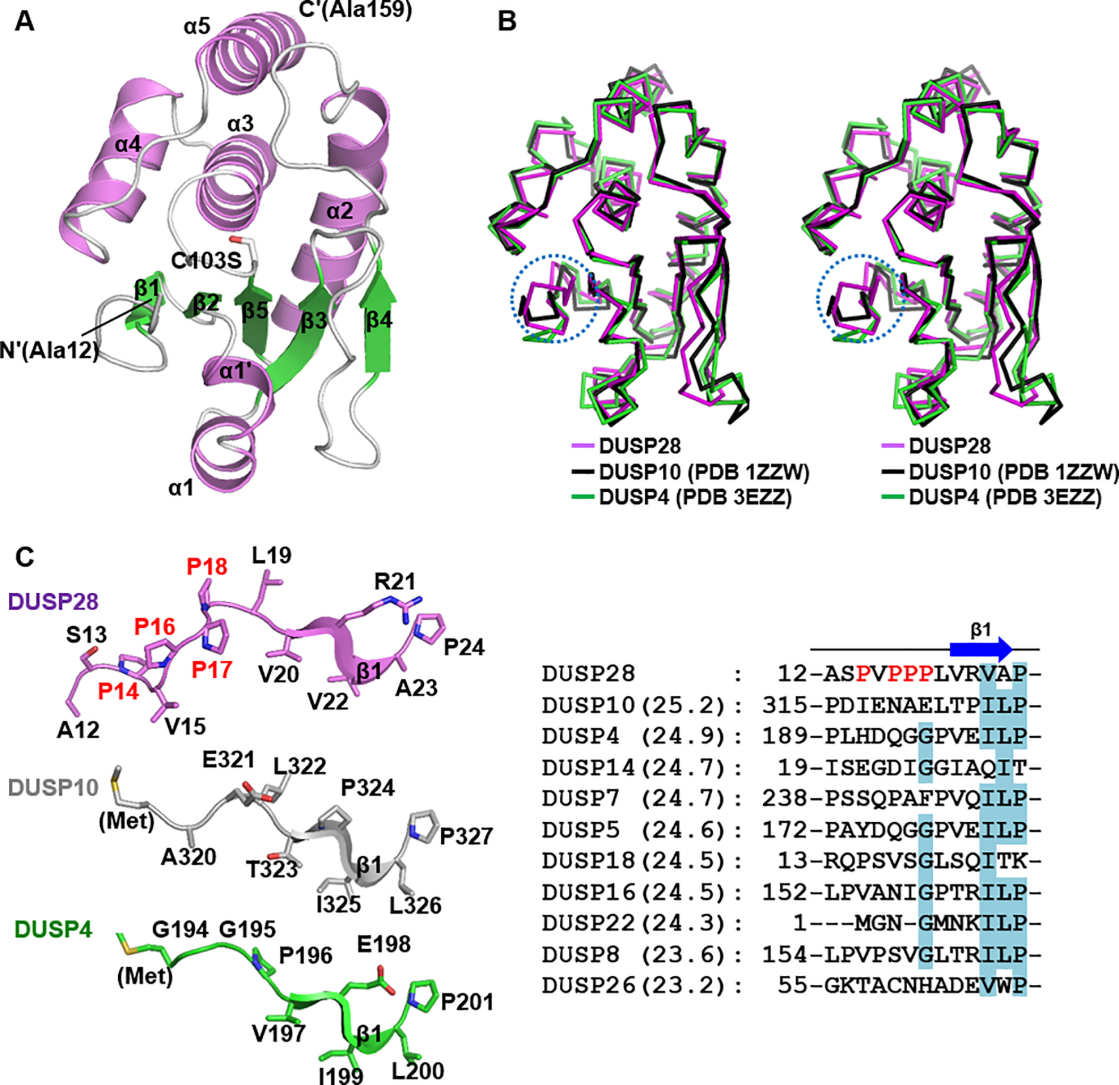
Crystal structure of human DUSP28. (A) DUSP28 is presented as secondary structure-labeled ribbon models, where α-helices are violet, β-strands are green, and the remaining structures are white. The serine-substituted catalytic residue is shown as a stick model and is labeled as “C103S”. (B) Stereo views of three superimposed DUSP proteins shown as C_α_ trace representation. Dashed circles indicate the DUSP28-specific N-terminal extension region. (C) The N-terminal region of DUSP proteins. (*Left*) N-terminal residues ahead of β1 from three DUSP proteins are presented as ribbon models and are labeled. (*Right*) Sequence alignment of N-terminal residues from DUSP28 and 10 other DUSP proteins ordered by decreasing Z-scores (noted in parenthesis). Four nonconserved proline residues in DUSP28 are labeled in red in both panels.

The DUSP28 monomer adopts a canonical DUSP fold composed of the core region (β1–β5 and α2–α5) and two mid-domain variations: α1' and α1 [6]. Five β-strands [β1(Val20−Ala23), β2(Leu26−Gly29), β3(Val45−Asn50), β4(Ala64−Arg67), and β5(Ala98−Tyr102)] constitute a central β-sheet, one side of which is surrounded by two α-helices [α1'(Ala31−Gly35) and α1(Glu37−Arg42)], and the other side of which is covered by four helices [α2(Leu79−Arg94), α3(Arg109−His121), α4(Leu126−Ala136), and α5(Pro144−Gln158); Fig 1A]. Our human DUSP28 structure is well matched to that of mouse DUSP28 when superimposed, with an RMSD of 0.85 Å over 143 aligned residues (not shown). A number of human PTP proteins were also identified in the search for homologous structures on the Dali server [21], including slingshot homolog 2 (PDB code 2NT2; Z-score 26.0), DUSP10 (PDB code 1ZZW; Z-score 25.3), and DUSP4 (PDB code 3EZZ; Z-score 25.0). Structural alignment between DUSP28 and two homologous DUSP proteins revealed that the structure of DUSP28 can be superimposed well on that of DUSP10 or DUSP4 with an RMSD of 1.10 Å over 143 aligned residues or 1.32 Å over 142 aligned residues (Fig 1B). One unique structural feature of DUSP28 is the presence of the proline-rich N-terminal extension region (residues 12−18 of DUSP28; A^12^SPVPPP^18^) immediately before β1 (Fig 1C, left); this unique proline-rich region is absent in homologous DUSP proteins (Fig 1C, right).

### Composition of the active motif of DUSP28

**Fig 2:**
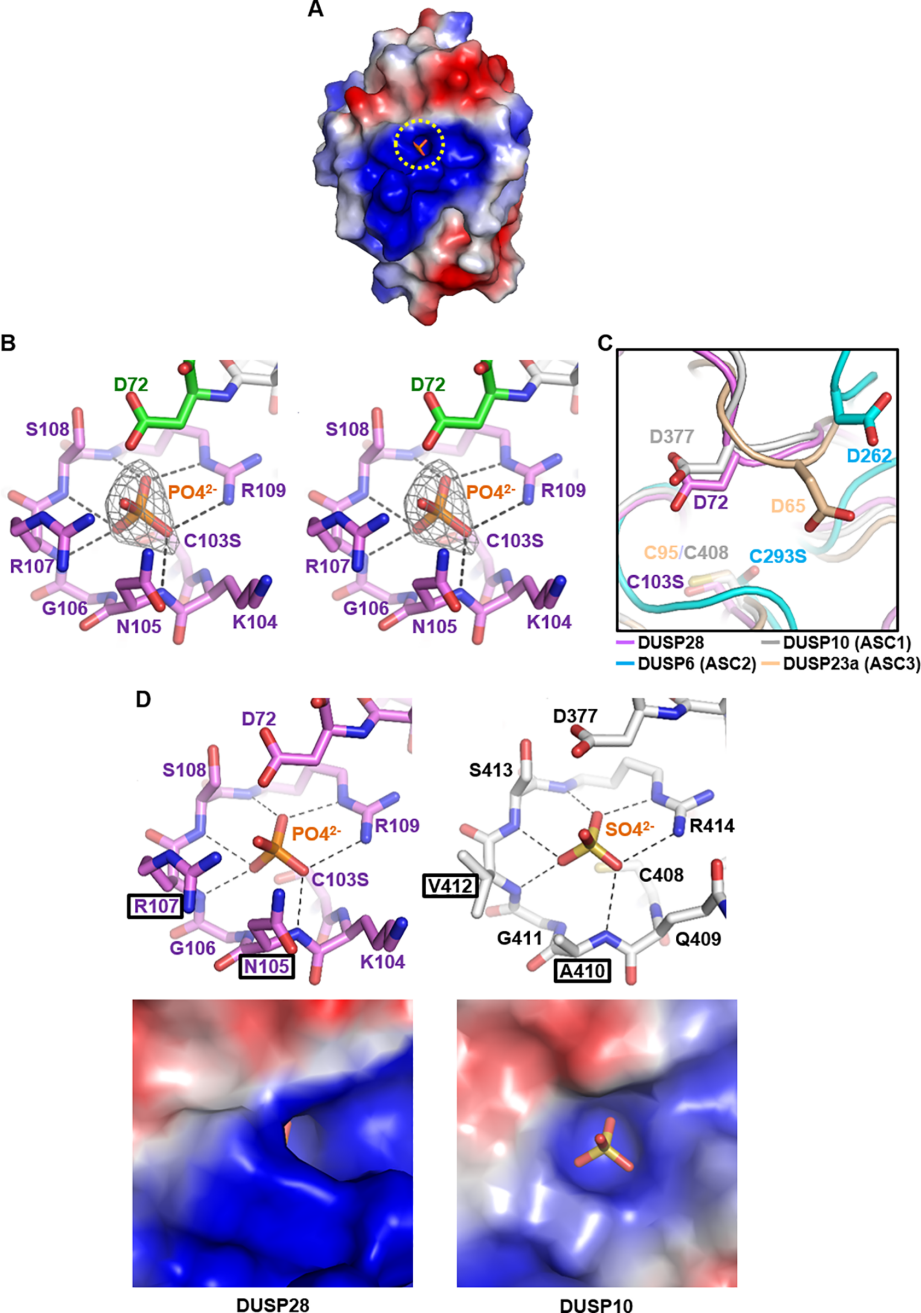
Structural analysis of the active site of DUSP28. (A) DUSP28 is shown as electrostatic surface representation together with a phosphate ion presented as sticks in a dashed circle. (B) Stereo views of the active site of DUSP28. Residues constituting the P-loop are shown violet, and a general acid/base residue Asp72 is green. Electrostatic interaction and hydrogen bonds mediated by a phosphate ion are presented as dotted lines. A 2Fo-Fc electron density omit map of the phosphate ion contoured at 5.0 σ is shown also. The temperature factor of the phosphate ion is 34.7. (C) ASC types. Four DUSP proteins are structurally aligned, representing their catalytic residues and general acid/base residues as sticks with labels. PDB codes are 1ZZW for DUSP10, 1MKP for DUSP6, and 2IMG for DUSP23a. (D) Structural comparison with the sulfate ion-bound active site of DUSP10. Active sites of two DUSP proteins are shown as sticks (top) or as electrostatic surface representation (bottom). Anions bound to the active sites are also presented as sticks. Rectangles indicate positions of two nonconserved residues in the PTP signature motif of DUSP28 and those of the corresponding residues of DUSP10.

In our human DUSP28 structure, the predominantly positively charged catalytic site pocket is occupied by a phosphate ion (Fig 2A). This anion is stabilized by a number of hydrogen bonds mediated by its oxygen atoms’ interactions with the backbone amides of β5−α3 loop (P-loop) residues including Asn105, Arg107, Ser108, and Arg109, and also by electrostatic interaction with the guanidinium group of Arg109 (Fig 2B). The PTP signature motif of DUSP28 (YC^103^KNGRSR^109^) contains the catalytic cysteine residue Cys103 (substituted with serine in our crystal structure) acting as a nucleophile in the dephosphorylating reaction as well as the conserved arginine residue Arg109 assisting the enzymatic reaction by anchoring the phosphate group of the substrate (Fig 2B). DUSP28 P-loop adopts a typical arrangement commonly found in catalytically active DUSP proteins; partially positively charged backbone amides as well as a guanidinium group of the conserved arginine residue (Arg109) are inwardly oriented towards the active site pocket, and therefore they create a positive electrostatic potential. It is a well-known critical factor for the enzyme activity of PTP proteins, as such a potential not only is complementary to the phosphate group from a substitute, but also reduces the p*K*a value of the catalytic cysteine residue for stabilizing it as a thiolate form [22–24]. The P-loop is located directly ahead of α3 in DUSP28, and thus α-helix dipole moment also contributes to the creation of the positive electrostatic potential [22]. Next, another conserved key residue for the PTP activity is the β4−α2 loop-located general acid/base aspartate residue (Asp72 in DUSP28; Fig 2B), which stabilizes the phosphoryl-intermediate during the enzymatic reaction. It is known that the conformation of β4−α2 loop, usually called the D-loop, determines the active site conformation (ASC) of DUSP proteins, which is classified into three types: ASC1 (closed state) and ASC2−3 (open state) [6]. We thus superposed our DUSP28 structure onto those of DUSP10, DUSP6, and DUSP23a, each of which respectively represents the three ASC types. As shown in Fig 2C, the general acid/base aspartate residue of DUSP28 is oriented toward the phosphate ion and the catalytic residue, as is that of DUSP10. Therefore, our DUSP28 structure is in the “closed” state (ASC1), like most previously determined DUSP structures, indicating that the composition of the D-loop and P-loop of DUSP28 is well-organized to serve its dephosphorylating activity [6].

**Fig 3:**
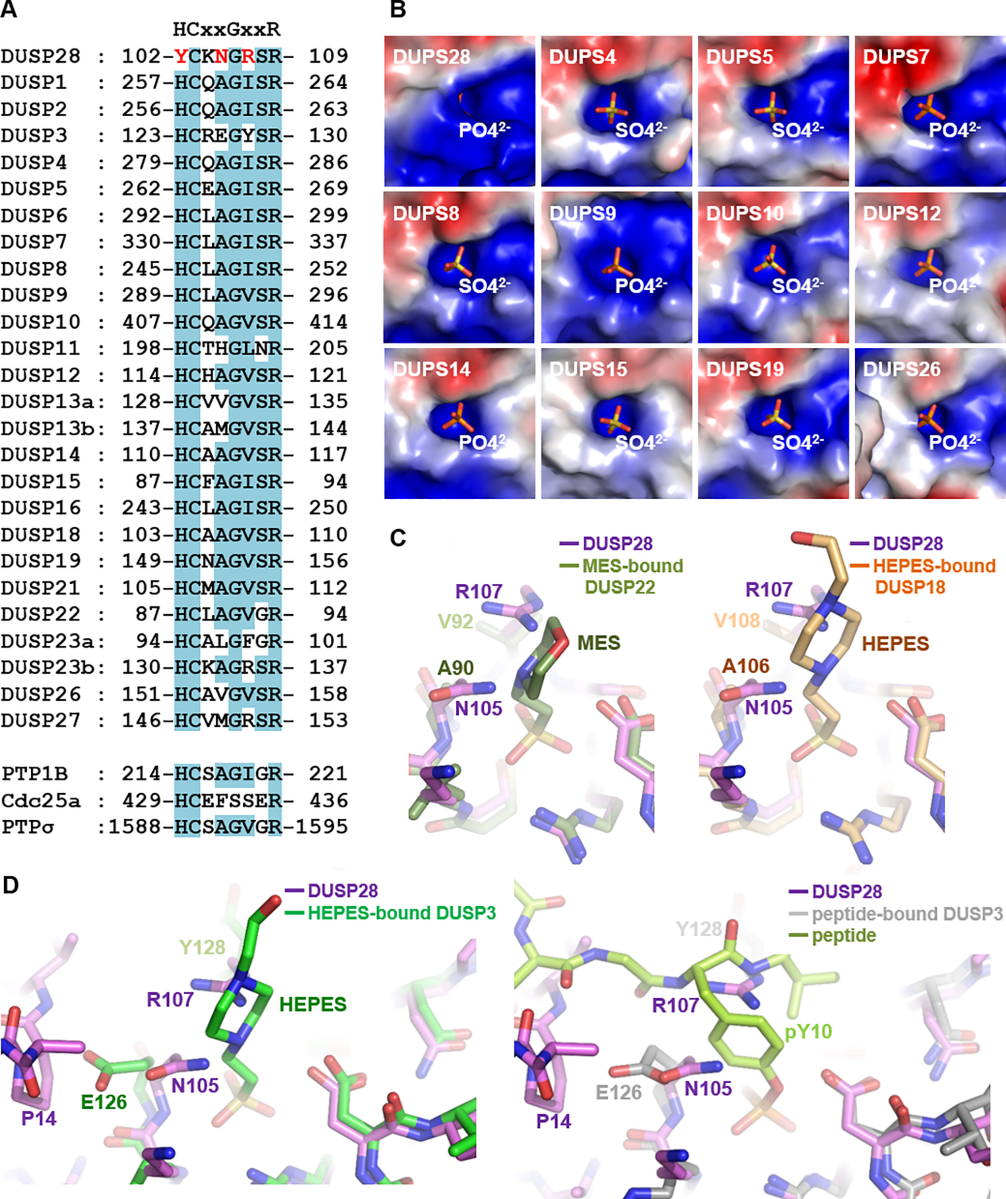
Effects of nonconserved residues in the P-loop of DUSP28. (A) Sequence alignment. The sequences of the P-loop of DUSP members and three non-DUSP PTPs are aligned with the PTP signature motif shown at the top. Conserved residues are shaded in cyan. Three nonconserved residues in the P-loop of DUSP28 are marked in red. (B) The active site pocket of twelve DUSP proteins are shown as electrostatic surface representation. Anions bound to the active sites are presented as sticks. (C and D) Structural alignment. The P-loop region of DUSP 28 is superimposed onto that of phosphotyrosine mimetic-bound DUSP22 (*C*, left; PDB code 1WRM) or DUSP18 (*C*, right; PDB code 2ESB), or onto that of DUSP3 bound to HEPES (*D*, left; PDB code 1VHR) or a peptide (*D*, right; PDB code 1J4X). The fourth and sixth residues in the PTP signature motif of the indicated DUSP proteins are labeled, together with DUSP28’s Pro14 residue, which is located in the N-terminal extension region of the protein (see Fig 1C).

Next, we structurally compared the P-loop of DUSP28 with that of DUSP10, which harbors a sulfate ion at the phosphate-binding site. At a glance, the two active motifs share high similarity in the backbone conformation, and they were indeed superposed well with each other, with an RMSD of 0.38 Å over 8 aligned C_α_ atoms (Fig 2D, top). Nevertheless, a significant difference between the two structures was observed; the side chains of Asn105 and Arg107 of DUSP28 are bulkier than the corresponding residues Ala410 and Val412 of DUSP10 (Fig 2D, top). This situation makes the active pocket of DUSP28 only partially exposed (Fig 2D, bottom). Asn105 and Arg107 of DUSP28 correspond to the fourth and sixth residues of the PTP signature motif HCxxGxxR. Alignment of the signature motifs from the other human DUSP proteins indicates that these positions are most conserved: alanine (in 18 of 26 DUSP proteins) and valine, leucine, or isoleucine (in 21 of 26 DUSP proteins; Fig 3A). To understand the effect of the presence of bulker residues at these positions, we first structurally compared the active site pocket of DUSP28 with those 11 DUSP that are bound to a phosphate or sulfate ion. Fig 3B shows that DUSP proteins commonly have an open and easily accessible active site pocket, except for DUSP28. Next, we structurally aligned our DUSP28 structure with two DUSP structures that are bound to 2-(N-morpholino)-ethanesulfonic acid (commonly called MES) or 4-(2-hydroxyethyl)-1-piperazineethanesulfonic acid (commonly called HEPES), respectively, both of which are known as phosphotyrosine mimetics. In the superposed models, side chains of Asn105 and Arg107 of DUSP28 cause steric hindrance with both MES (Fig 3C, left) and HEPES (Fig 3C, right). After that, our DUSP28 structure was superimposed onto two DUSP3 structures that are bound to either HEPES or a phosphotyrosine-containing peptide. It was confirmed that similar steric hindrance also takes place between the two DUSP28 residues and HEPES (Fig 3D, left) or phosphotyrosine (Fig 3D, right). The fourth residue in the PTP signature motif of DUSP3 is glutamate (Glu126), which is actually bulkier than asparagine in terms of side chain size. Nonetheless, although the side chain in Glu126 of DUSP3 is directed to the outside of the active site, that of Asn105 in DUSP28 is not, presumably owing to the presence of a proline residue (Pro14) that is contained in the DUSP28-specific N-terminal extension region (Fig 3D). Thus, the active site pocket of DUSP28 is only partially exposed, in the presence of Asn105 and Arg107, which can prevent phosphotyrosine from being accommodated. Presumably, this active site pocket is hardly accessible to a substrate as compared to those of other DUSP proteins. We therefore hypothesized that the dephosphorylating activity of DUSP28 is lower than that of other active DUSP subfamily members, and can be improved by introducing mutations into the two amino acid positions of DUSP28.

### Exceptionally low phosphatase activity of DUSP28

**Fig 4:**
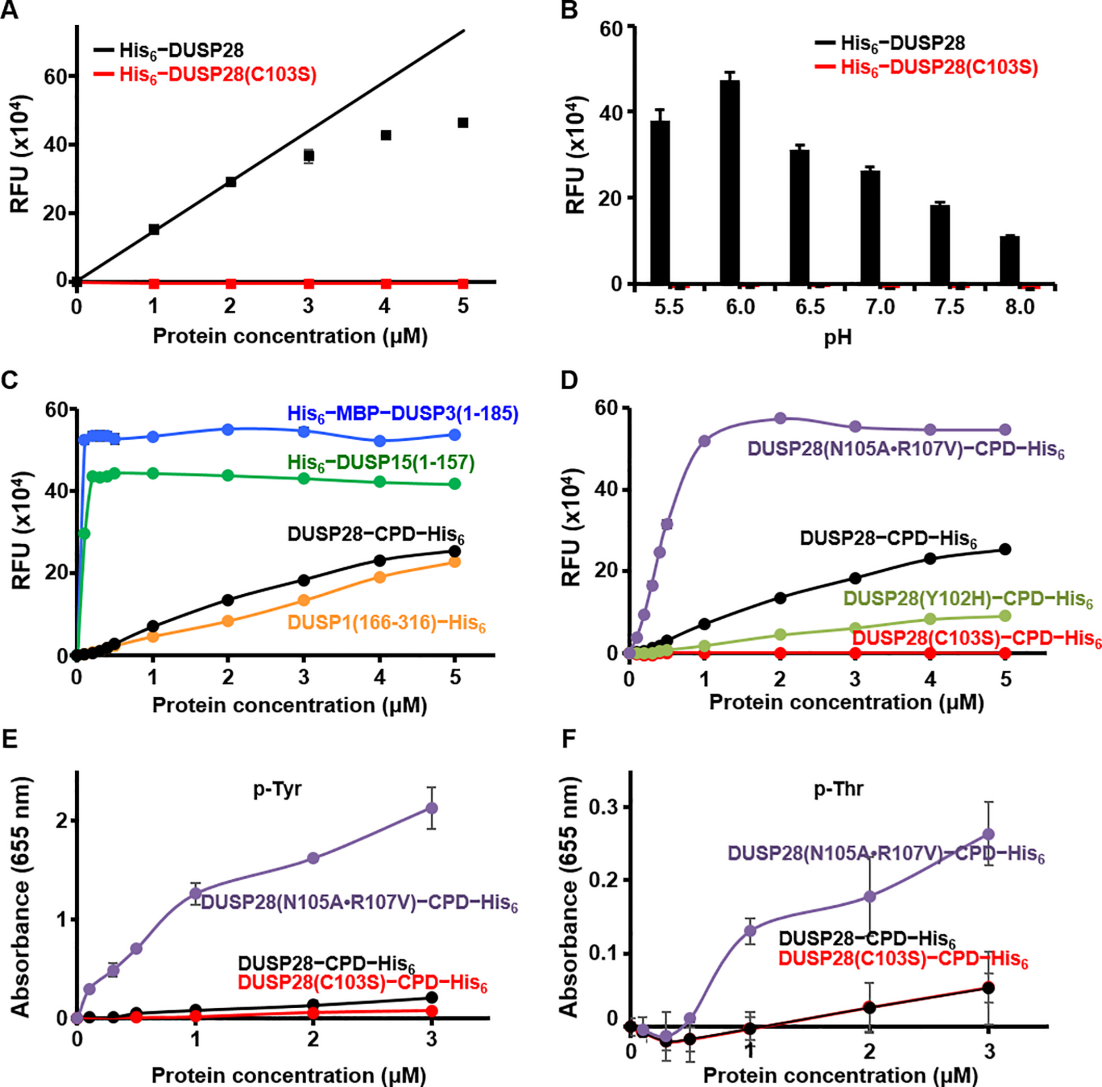
Characterization of phosphatase activity of DUSP28. Enzymatic assays were performed as described in the Materials and Methods section. RFU stands for relative fluorescence units. pH was 6.0 in *A* and *C*−*F*, and as indicated in panel *B*. Protein concentrations were 2 μM in *B*, and as indicated in *A* and *C*−*F*. Substrates were DuFMUP in *A*−*D* and indicated phosphoamino acids in *E* and *F*. (A and B) Phosphatase activities of wild-type and C103S mutant DUSP28 proteins were assayed at various protein concentrations (*A*) or at various pH levels (*B*) and were compared. (C and D) Enzymatic activities of four DUSP proteins (DUSP1, DUSP3, DUSP15, and DUSP28) and three kinds of DUSP28 mutants were assayed at various protein concentrations and were compared. CPD indicates the cysteine protease domain of the *Vibrio cholera* MARTX toxin protein. It was fused with DUSP28 proteins to improve protein solubility and stability of DUSP28(N105A•R107V) and DUSP28(Y102H) that severely precipitated during purification without tagging with the CPD protein. DUSP28 and DUSP28(C103S) were also tagged with the identical tag for comparing their activity in the same condition. (E and F) Dephosphorylating activities of three DUSP28 constructs toward phosphoamino acids. Phosphotyrosine and phosphothreonine are denoted as p-Tyr and p-Thr, respectively. Phosphatase activities were measured using the malachite green phosphate assay kit as described in Material and Methods section in detail.

To test this hypothesis, *in vitro* biochemical assays were performed to assess the dephosphorylating activity of DUSP28. First, phosphatase activities of wild-type DUSP28 and of the catalytically inactive DUSP28(C103S) mutant were evaluated using 6,8-difluoro-4-methylumbelliferyl phosphate (DiFMUP), *para*-nitrophenylphosphate (*p*NPP), or each of three phosphoamino acids (phosphoserine, phosphothreonine, or phosphotyrosine) substrate. As shown in Fig 4A, free phosphate was detected dose-dependently in the reaction mixture containing wild-type DUSP28−but not DUSP28(C103S)−when DiFMUP was used as a substrate, indicating that DiFMUP was hydrolyzed by the enzymatically active form of DUSP28. The phosphatase activity of DUSP28 was optimal at pH 6.0 (Fig 4B). We tried to calculate kinetic constants of the DUSP28 dephosphorylating reactions without success. The reason is that the initial velocity data required for calculating V_max_, *k*_M_, and *k*_cat_ could not be obtained because the enzymatic reaction of DUSP28 was unsaturated despite a number of trials at different time scales and at various protein and substrate concentrations (data not shown), indicating that the phosphatase activity of DUSP28 is exceptionally low. Moreover, dephosphorylating activity of DUSP28 was not detected when *p*NPP or three phosphoamino acids were used as substrates despite repeated trials under a variety of reaction conditions (data not shown). We thus attempted to demonstrate the low activity of DUSP28 by comparing it with the activity of other DUSP proteins. Fig 4C clearly shows that the phosphatase activity of DUSP28 is much lower than those of DUSP3 and DUSP15, which are well-characterized DUSP enzymes having a potent dephosphorylating activity [25,26]. On the other hand, the enzymatic activity of DUSP28 is comparable with that of DUSP1 (also known as MKP-1), which belongs to the MKP subfamily and is known to be weakly active by itself but to be robustly activated by interaction with MAPKs, such as extracellular signal-regulated kinase 2, c-Jun N-terminal kinase 1, and p38α, through its MKB domain [27]. It has also been reported that several other DUSP proteins in the MKP subfamily, e,g., DUSP4, DUSP6, and DUSP9 (also known as MKP-2, MKP-3, and MKP-4, respectively), get activated via a similar mechanism [28–32]. DUSP28, however, is not classified as the MKP subfamily member and contains neither an MKB domain nor any other domain except for the phosphatase domain, suggesting that this weak activity may be a specific and novel feature of DUSP28 differentiating this protein from all other DUSP proteins.

Next, a mutant DUSP28 protein harboring mutations Asn105Ala and Arg107Val within the PTP signature motif was prepared, and its dephosphorylating activity was compared with that of wild-type DUSP28. As shown in Fig 4D, the enzymatic activity of DUSP28 toward DiFMUP was remarkably improved by these mutations and became comparable with the activities of DUSP3 and DUSP15 (Fig 4C). This mutant protein also showed a remarkably elevated dephosphorylating activity toward phosphotyrosine (Fig 4E) and a moderately enhanced activity toward phosphothreonine (Fig 4F) when compared to wild-type DUSP28 and catalytically inactive DUSP28. We note that all the three proteins did not exhibit a notable activity toward phosphoserine (not shown). These results clearly indicate that the weak activity of DUSP28 is mostly explained by the low accessibility of its catalytic pocket (see Fig 2D) because of the presence of nonconserved asparagine and arginine residues in the P-loop region (see Fig 3).

### DUSP28 contains tyrosine instead of histidine immediately before active cysteine

**Fig 5:**
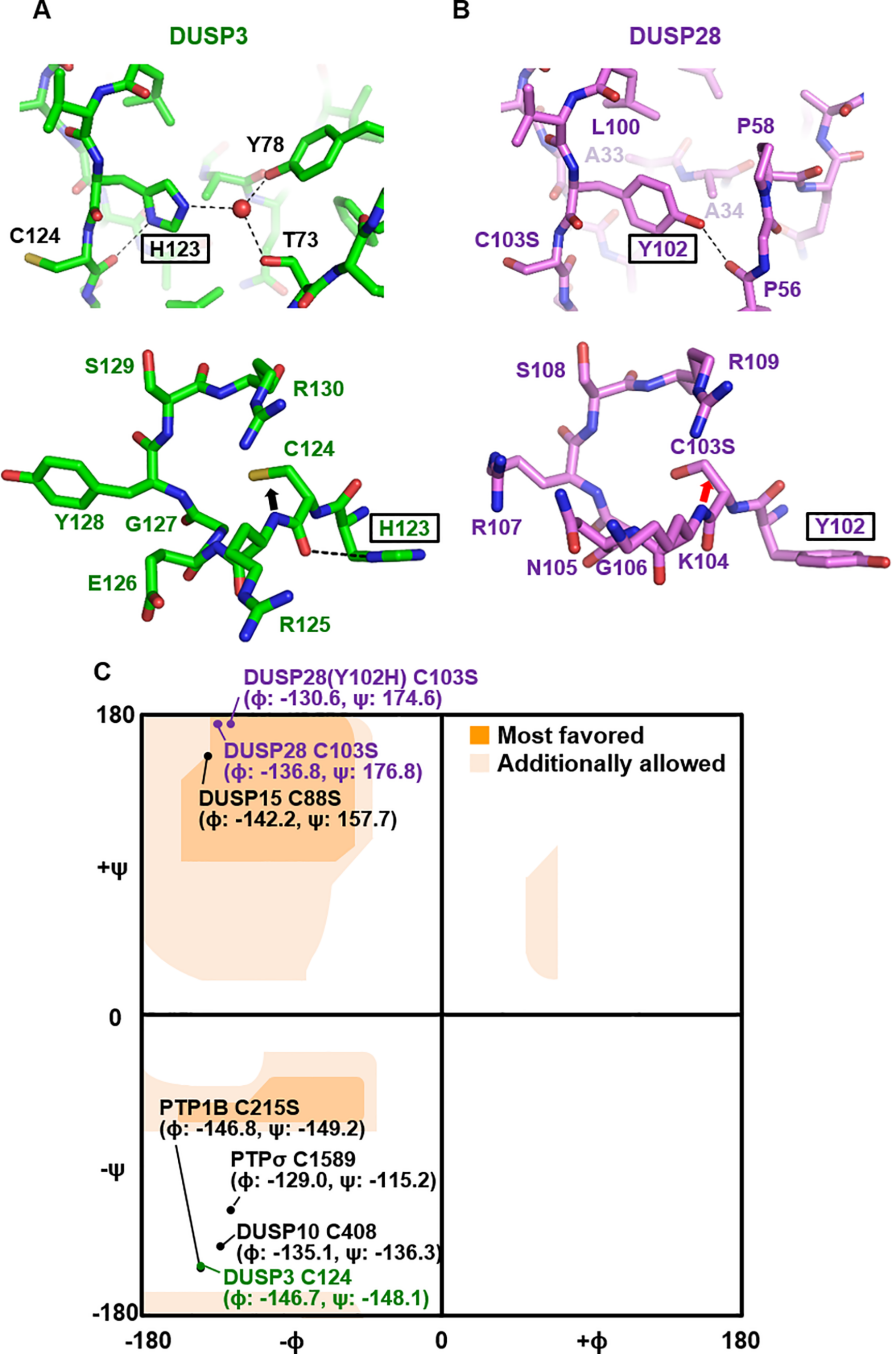
Structural analysis of the atypical Tyr102 residue in DUSP28. (A and B) Structural roles of His123 in DUSP3 (*A*) and of Tyr102 in DUSP28 (*B*) are aligned and compared. Dashed lines indicate hydrogen bonds associated with those residues. A water molecule involved in the DUSP3 His123-mediated hydrogen bond network is shown as a red sphere (*A*, top). Directions of the backbone amide of the catalytic site residue are highlighted by arrows in both structures (*A* and *B*, bottom). (C) ϕ and ψ dihedral angles of the catalytic residue of five DUSP proteins and two non-DUSP proteins are presented on the Ramachandran plot. Extended and detailed information about the other members of the DUSP subfamily is shown in S3 Fig.

Another unique feature of the PTP signature motif of DUSP28 is that the residue immediately preceding the catalytic cysteine is tyrosine, in contrast to all the other human DUSP proteins, which contain a histidine residue at this position (Fig 3A). The role of the conserved histidine residue has been studied in DUSP3 [33]. Through its imidazole ring, this residue mediates an array of hydrogen bonds (Fig 5A, top), which were revealed to be crucial for the proper positioning of the catalytic cysteine residue and the enzymatic activity of DUSP3, because the phenylalanine substitution of DUSP3 His123 results in a 19-fold decrease in the *k*_cat_/*K*_M_ value, from 835 to 43 s^-1^ M^-1^ [33]. Particularly, the N_δ_ atom of this invariant histidine attracts the main chain carbonyl group of the active cysteine in DUSP3 via a hydrogen bond, thereby directing the backbone amide of the active cysteine to the active site pocket (Fig 5A bottom; indicated by a black arrow) and resulting in unfavorable ϕ and ψ dihedral angles of the catalytic residue (Fig 5C). This is one of the well-known characteristics, present in most PTP proteins [34]. A similar P-loop arrangement was detected in a variety of PTP proteins such as DUSP10, DUSP19, DUSP26, PTP1B, PTPσ and Cdc25a (S2 Fig). In contrast, Tyr102 of DUSP28 cannot form a hydrogen bond with the main chain carbonyl group of the active site residue (Fig 5B, bottom). Instead, it appears to play a structural role in sustaining the protein folding through hydrophobic interactions with neighboring residues such as Ala33, Ala34, Pro58, and Leu100 and via a hydrogen bond between its hydroxy group and the main chain carbonyl of Pro56 (Fig 5B, top). In agreement with these data, the direction of the backbone amide of C103S in DUSP28 is toward the outside of active site pocket (Fig 5B, bottom), and its ϕ and ψ dihedral angles are in the “most favored” region, unlike those of DUSP3, DUSP10, PTP1B, and PTPσ that are in the “unfavorable” region, on the Ramachandran plot (Fig 5C and S3 Fig).

Dihedral angles of catalytic residues of 21 DUSP proteins and three non-DUSP PTP proteins were examined and compared next (S3 Fig). We found that ϕ and ψ values of the catalytic residue of several MKP subfamily members, such as DUSP6 and DUSP9, are located in the “most favored” region of the Ramachandran plot, as is that of DUSP28 (S3 Fig). It therefore appears to be clear that the ϕ and ψ dihedral angles of the catalytic cysteine residue is directly associated with the presence or absence of the histidine−main chain attraction in DUSP proteins(S3 Fig). Nevertheless, whether enzymatic activity of a DUSP protein is determined by the direction of a single backbone amide is questionable. The previously reported low enzymatic activities of DUSP6 and DUSP9 can be explained by the overall impairment in the conformation of the P-loop rather than by a single amino acid distortion (S2 Fig). Furthermore, DUSP15, whose catalytic residue is positioned near that of DUSP28 on the Ramachandran plot (Fig 5C), showed a robust activity in a dephosphorylation assay (Fig 4C), despite a lack of the imidazole group-mediated main chain carbonyl-attracting hydrogen bond (S2 Fig).

### Determination of crystal structure and enzymatic property of DUSP28(Y102H)

To verify whether substitution of Tyr102 with a canonical histidine residue can cause structural changes to DUSP28 and improve its phosphatase activity, a mutant DUSP28 protein containing the Tyr102His mutation, referred to as DUSP28(Y102H), was prepared and subjected to crystallization. We obtained DUSP28(Y102H) crystals in the nearly the same condition to that of DUSP28 crystals (see Materials and Methods), and its structure was finally determined to 2.4 Å resolution (Table 1). Although the crystal structure of DUSP28(Y102H) was fairly well matched to that of DUSP28 when superimposed, with an RMSD of 0.50 Å over 147 aligned residues (S4A Fig), the Tyr102His substitution was revealed to result in noticeable conformational change of residues Ala33−Ala34 and Gly57−Pro61 (S4B Fig). These residues are in contact with Tyr102 in the original crystal structure (Fig 5B and S4B Fig), clearly supporting the structural role of Tyr102 in sustaining the DUSP28 protein fold. Nevertheless, the main chain carbonyl-attracting hydrogen bond is not shown in the DUSP28(Y102H) structure as was in the DUSP28 structure (S4B Fig), consistently with the nearly identical dihedral angles of the C103S residue from DUSP28 and DUSP28(Y102H) (Fig 5C and S3 Fig). Next, the enzymatic activity of DUSP28(Y102H) was examined and compared with that of wild-type DUSP28. As shown in Fig 4D, the phosphatase activity of DUSP28 was rather reduced by the Tyr102His mutation, in contrast to the introduction of the Asn105Ala and Arg107Val double-mutation, which considerably improved the enzymatic activity irrespective of the presence of Tyr102. In conclusion, DUSP28 contains a tyrosine instead of histidine at the position ahead of the active site residue, and this tyrosine may play a structural role. On the other hand, this substitution may not be the key determinant of the atypically low activity of DUSP28.

## Discussion

In this work, we determined the crystal structures of two forms of human DUSP28, and carried out detailed structural and biochemical analyses of this protein. Our crystal structure shows that DUSP28 adopts a canonical DUSP fold overall, in which a central β-sheet is sandwiched by α-helices on the two sides, and that this protein contains a proline-rich N-terminal extension region (Fig 1). To the authors' surprise, DUSP28 has an only partially exposed active site pocket (Figs 2 and 3) and exceptionally low phosphatase activity (Fig 4), which together differentiate this protein from other DUSPs. A unique PTP signature motif of DUSP28 contains three characterized residues: Tyr102, Asn105, and Arg107, which are most conserved as histidine, alanine, and valine/leucine/isoleucine, respectively, in most DUSP members (Fig 3A). Enzymatic activity assays showed that the phosphatase activity of DUSP28 can be considerably improved by the Asn105Ala and Arg107Val double-substitution (Fig 4D, 4E and 4F), indicating that the low dephosphorylating activity of DUSP28 must have resulted from the substrate accommodation hindrance by the side chains of Asn105 and Arg107. To the best of our knowledge, it is a novel and unique mechanism for the regulation of enzymatic activity among the DUSP subfamily proteins. We further suppose a possibility that DUSP28’s distinctive active site loop may specify the substrate recognition of this enzyme; DUSP28 may get activated by a protein-protein interaction that induces a reorientation of side chains of Asn105 and Arg107 and an improvement of accessibility of the catalytic cysteine residue, similarly to the MKP subfamily members such as DUSP1 and DUSP4 that get activated by the MAPK interaction-mediated P-loop rearrangement [27–32]. We consider that verification of the physiological function and significance of the phosphatase activity of DUSP28 in cells as well as identification of DUSP28-interacting proteins, which remain to be elucidated, will be necessary for the determination of the substrate specification issue and for the enlargement of our understanding of DUSP28.

Herein, we provided the structural and biochemical data of DUSP28, which will serve as a basis not only for further researches aimed at unraveling the precise function and mechanism of action of DUSP28, but also for investigations of small molecules targeting this enzyme in the therapeutic approach. These works will be worthwhile because of recent reports indicating DUSP28’s involvement in hepatocellular and pancreatic cancers [16–18].

## Materials and methods

### Preparation, crystallization, and determination of structure of DUSP28

The DNA fragment coding for human DUSP28 was amplified by polymerase chain reaction and cloned into the pET28a plasmid (Novagen), which was served as a template for preparation of the DUSP28 construct containing substitutions R59Q and C103S. The R59Q mutation was introduced to remove a thrombin cleavage site within the protein, whereas the C103S mutation was introduced to abrogate DUSP28’s catalytic activity, which may hamper this protein’s crystallization. The DUSP28(Y102H) construct was also prepared by additionally introducing Y102H mutation into the construct. Each of the DUSP28 proteins was produced in the *Escherichia coli* BL21(DE3) strain (Novagen) at 18°C and was initially purified on a HiTrap TALON crude column (GE Healthcare). After treatment with thrombin protease (Haematologic Technologies) to cleave the N-terminal His_6_-tag, the proteins were further purified on a HiPrep 26/60 Sephacryl S-100 HR gel filtration column (GE Healthcare) equilibrated with running buffer (20 mM Tris-HCl pH 8.0, 500 mM NaCl, and 4 mM dithiothreitol). Crystals were obtained by the sitting-drop vapor diffusion method at 18°C by mixing and equilibrating 0.2 μL samples of the protein solution (20 mg/mL) and a precipitant solution (100 mM Tris-HCl pH 8.5, 300 mM NaCl, and 26% [w/v] polyethylene glycol 3350 for DUSP28 crystals; 100 mM Tris-HCl pH 8.5, 100 mM NaCl, and 28% [w/v] polyethylene glycol 3350 for DUSP28(Y102H) crystals). Before data collection, the crystals were immersed briefly in a cryoprotectant solution, which was the reservoir solution with 20% glycerol. Diffraction data were collected on the beamline 7A at the Pohang Accelerator Laboratory, Korea, and processed using the *HKL* 2000 software [35]. The structures were determined by the molecular replacement method in the Phaser software [36] using the structure of mouse DUSP28 (PDB code 2HCM) as a search model for determining the human DUSP28 structure, which was then used as a search model for determining the DUSP28(Y102H) structure. Software tools Coot [37] and PHENIX [38] were employed for the model building and refinement, respectively. Crystallographic data statistics are summarized in Table 1.

### SEC-MALS

Protein samples were diluted to a concentration of 2, 10, and 20 mg/mL in a buffer consisting of 20 mM Tris-HCl pH 7.5, 500 mM NaCl, 5% (v/v) glycerol, and 2 mM dithiothreitol). SEC-MALS was carried out using a Superdex^TM^ 75 10/300 GL column (GE Healthcare), DAWN HELEOS-II (Wyatt Technology Corporation), Optilab T-rEX (Wyatt Technology Corporation), and ASTRA version 6.1 (Wyatt Technology Corporation) coupled with high-performance liquid chromatography (Shimadzu).

### Preparation of proteins for an activity assay

Each of the DNA fragments coding for DUSP1 (residues 166−316) or DUSP15 (residues 1−157) was cloned into plasmids pET21a (Novagen) and pET28a, respectively. The DNA fragment coding for DUSP3 (residues 1−185) was cloned into the modified pET28a plasmid designed to tag the protein with N-terminally His_6_-linked maltose-binding protein (MBP). Wild-type and mutant DUSP28 proteins containing C103S, Y102H, or the N105A•R107V substitution were cloned into pET22b (Novagen) that was modified to tag the protein with a C-terminal His_10_-linked cysteine protease domain. These proteins were produced and purified using a procedure similar to that for the DUSP28(R59Q•C103S) enzyme.

### Dephosphorylation activity assays

Enzymatic assays toward 100 μM DiFMUP were conducted using 0−5 μM purified recombinant proteins at 37°C for 1 h in a 100 μL reaction mixture containing 50 mM NaCl, 2 mM dithiothreitol, and a 100 mM buffer that was used to adjust pH: to 5.5 with sodium citrate, to 6.0 and 6.5 with Bis-Tris, and to 7.0, 7.5, and 8.0 with Tris-HCl. Enzymatic activities were measured in triplicate for all the substrates by detecting the reaction product as DiFMU-dependent relative florescence units by monitoring the excitation/emission at 355/450 nm on the multimode plate reader Victor X3 (Perkin Elmer). Enzymatic assays toward 2 mM phosphoamino acids were performed using 0−3 μM purified recombinant proteins at 37°C for 1 h in an 80 μL reaction mixture containing 100 mM Bis-Tris (pH 6.0), 50 mM NaCl, and 2 mM dithiothreitol. The release of inorganic phosphate was detected using the malachite green phosphate assay kit (Bioassay Systems) in triplicate, and the absorbance at 655 nm was read 30 min later on the SpectraMax Plus 384 microplate reader (Molecular Devices). Control samples that did not contain any protein were used to detect nonenzymatic substrate hydrolysis for baseline correction. The reaction conditions were optimized by varying the reaction time, protein and substrate amounts, and pH.

### Accession numbers

The coordinates of DUSP28 and DUSP28(Y102H) together with the structure factors have been deposited in the Protein Data Bank under the accession code 5Y15 and 5Y16, respectively.

## Supporting information

S1 Fig Analysis of the stoichiometry of DUSP28. (A) (*Top*) Two DUSP28 molecules in an asymmetric unit are presented as ribbon models in violet and green, respectively. For clarity, secondary structures according to the order of their appearance in the primary sequence are labeled in molecule A only. (*Bottom*) Putative dimerization interface shown at top was analyzed using the PISA server. Δ^i^G, the solvation free energy gain upon formation of the interface; N_HB_, the number of potential hydrogen bonds across the interface; N_SB_, the number of potential salt bridges across the interface. (B) Native gel electrophoresis was performed under acidic conditions with reversed polarity due to the basic pI (8.27) of DUSP28 at neutral pH. 5 μg DUSP28 protein was mixed with 4x dissolving buffer containing 60 mM potassium acetate (pH 6.8), 37%(v/v) glycerol, and 0.05% methylene blue, and was loaded onto a 12% polyacrylamide gel. Electrophoresis was carried out in running buffer containing 140 mM acetic acid (pH 4.3) and 350 mM β-alanine at 120 V for 80 min at 277 K, which was then followed by Coomassie Blue staining. (C) SEC-MALS analysis. (*Top*) Molar mass (in kg/mol) and LS detector readings (in arbitrary units; a.u.) are plotted against the elution time (in min) of the size exclusion column. (*Bottom*) DUSP28 exists as a monomer, not a dimer, in solution. Conc., concentration; Mw, weight-average molar mass; Mn, number-average molar mass.
(TIF)

S2 Fig Active loop conformation of PTP proteins. PTP signature motifs from nine PTP proteins including six DUSPs are presented as stick models and are labeled. Dotted lines indicate hydrogen bonds between the main chain carbonyl group of the active site residue and the N_δ_ atom of the conserved histidine. Direction of the backbone amide of the active site residue is highlighted by arrows in each structure.
(TIF)

S3 Fig ϕ and ψ dihedral angles of the catalytic residue of PTP proteins. Main chain dihedral angles of the catalytic residues of 20 DUSPs and three non-DUSP PTPs (blue) are presented on the Ramachandran plot diagram. DUSP28 proteins are red, and the rest DUSPs within the favored or allowed region are green; all the other DUSP proteins are black. Detailed information about the 23 proteins is listed on the right. “O-N attraction” indicates the presence of the attraction between the histidine residue ahead of the catalytic cysteine and the main chain carbonyl group of the catalytic residue.
(TIF)

S4 Fig Structural comparison of DUSP28 and DUSP28(Y102H). (A) Stereo views of the superimposed structures of DUSP28 in violet and DUSP28(Y102H) in cyan shown as C_α_ trace representation. C103S and Y102H residues of DUSP28(Y102H) are presented as sticks with labels. (B) Stick models of DUSP28 and DUSP28(Y102H) are structurally aligned and compared. The substituted residues (highlighted by rectangles) as well as residues showing conformational alteration between the two structures are labeled. Distance between the N_δ_ atom of the substituted histidine residue and the main chain carbonyl group of the catalytic residue is presented with an arrow; it is longer than the limit of distance for an energetically significant hydrogen bond, 3.5 Å.
(TIF)
