# Supplementary figures and images for: Structural and biochemical analysis of atypically low dephosphorylating activity of human dual-specificity phosphatase 28
Source: PLoS One. 2017 Nov 9;12(11):e0187701. doi: 10.1371/journal.pone.0187701 (PMC5679558; doi:10.1371/journal.pone.0187701)

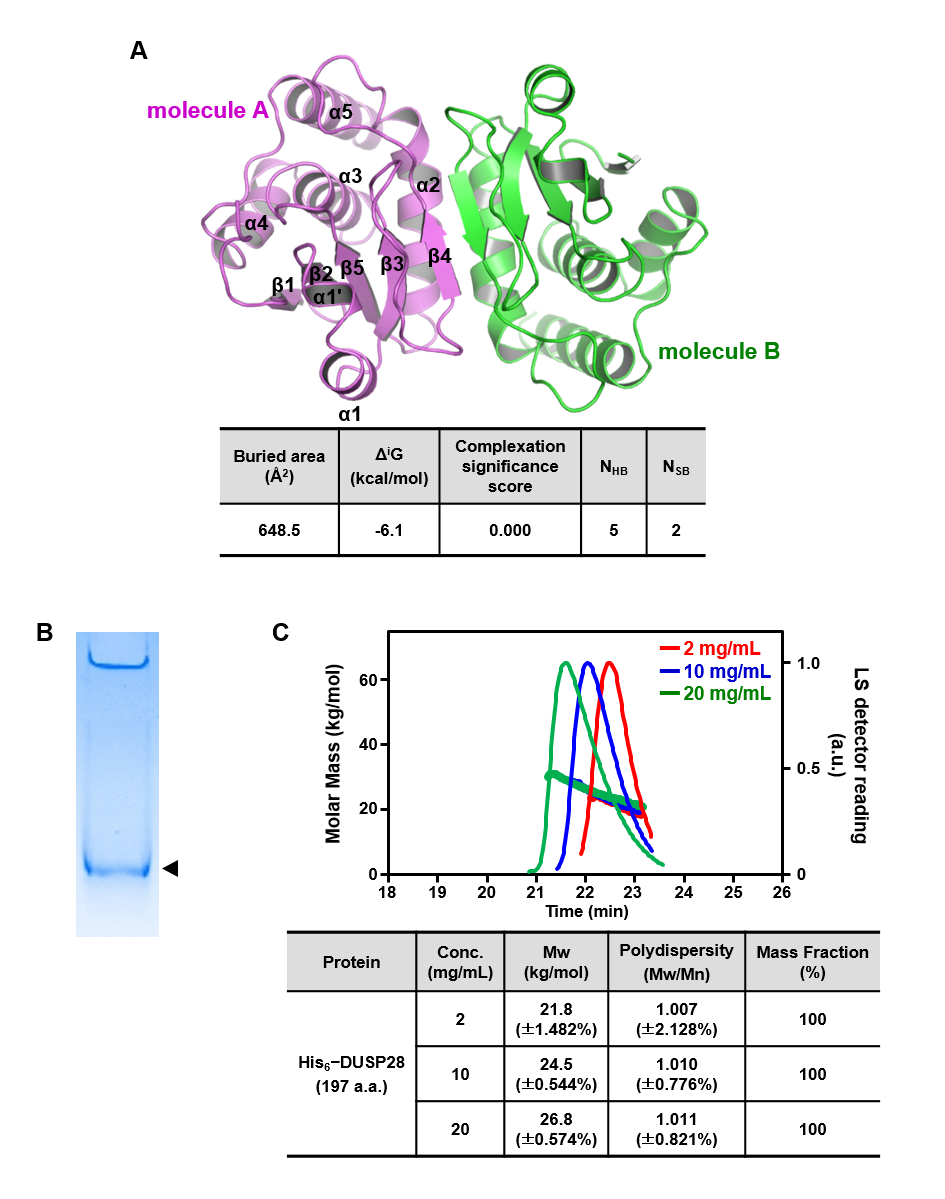

Supplement: S1 Fig — (A) (Top) Two DUSP28 molecules in an asymmetric unit are presented as ribbon models in violet and green, respectively. For clarity, secondary structures according to the order of their appearance in the primary sequence are labeled in molecule A only. (Bottom) Putative dimerization interface shown at top was analyzed using the PISA server. ΔiG, the solvation free energy gain upon formation of the interface; NHB, the number of potential hydrogen bonds across the interface; NSB, the number of potential salt bridges across the interface. (B) Native gel electrophoresis was performed under acidic conditions with reversed polarity due to the basic pI (8.27) of DUSP28 at neutral pH. 5 μg DUSP28 protein was mixed with 4x dissolving buffer containing 60 mM potassium acetate (pH 6.8), 37%(v/v) glycerol, and 0.05% methylene blue, and was loaded onto a 12% polyacrylamide gel. Electrophoresis was carried out in running buffer containing 140 mM acetic acid (pH 4.3) and 350 mM β-alanine at 120 V for 80 min at 277 K, which was then followed by Coomassie Blue staining. (C) SEC-MALS analysis. (Top) Molar mass (in kg/mol) and LS detector readings (in arbitrary units; a.u.) are plotted against the elution time (in min) of the size exclusion column. (Bottom) DUSP28 exists as a monomer, not a dimer, in solution. Conc., concentration; Mw, weight-average molar mass; Mn, number-average molar mass. (TIF) [file pone.0187701.s001.tif]

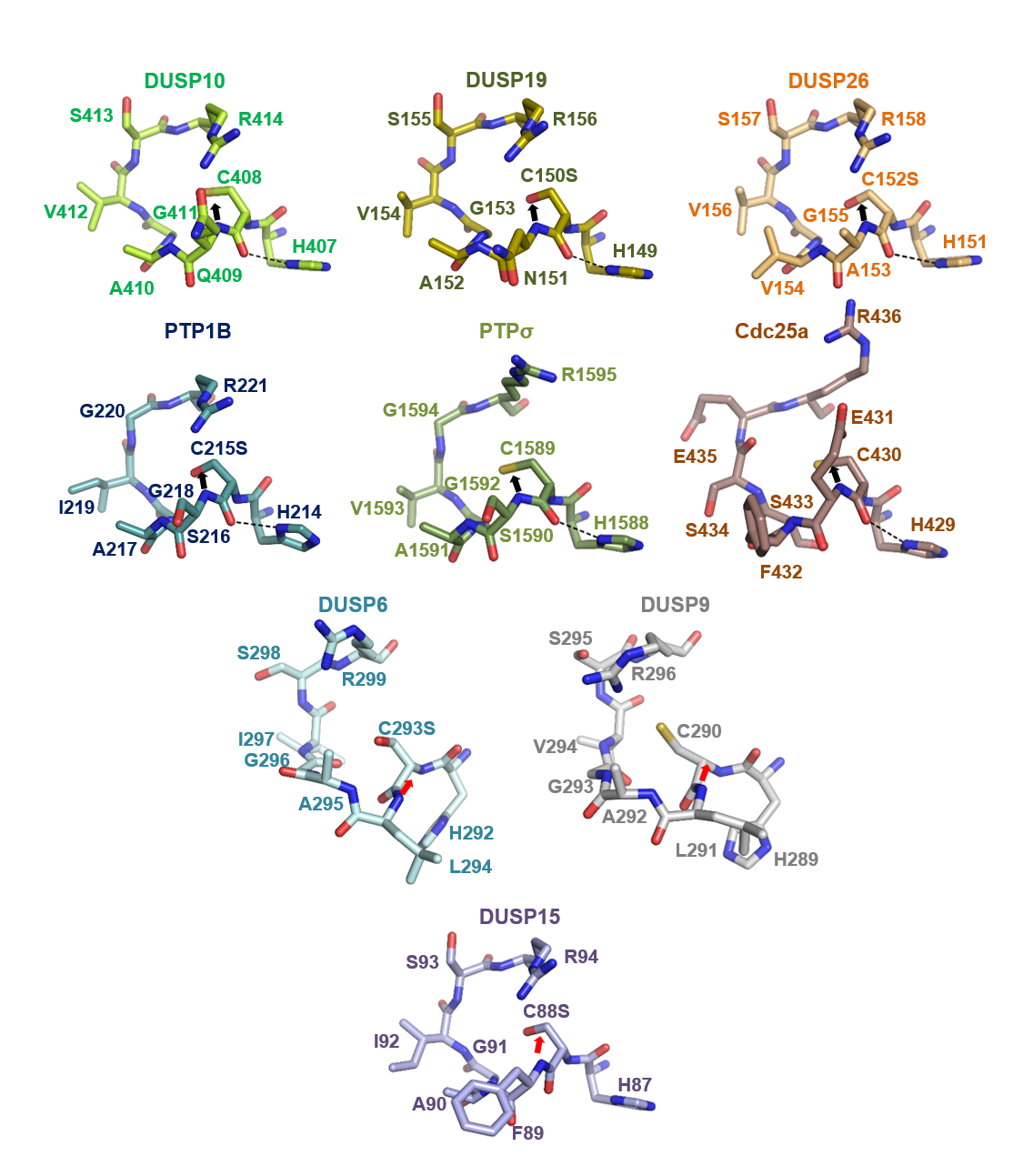

Supplement: S2 Fig — PTP signature motifs from nine PTP proteins including six DUSPs are presented as stick models and are labeled. Dotted lines indicate hydrogen bonds between the main chain carbonyl group of the active site residue and the Nδ atom of the conserved histidine. Direction of the backbone amide of the active site residue is highlighted by arrows in each structure. (TIF) [file pone.0187701.s002.tif]

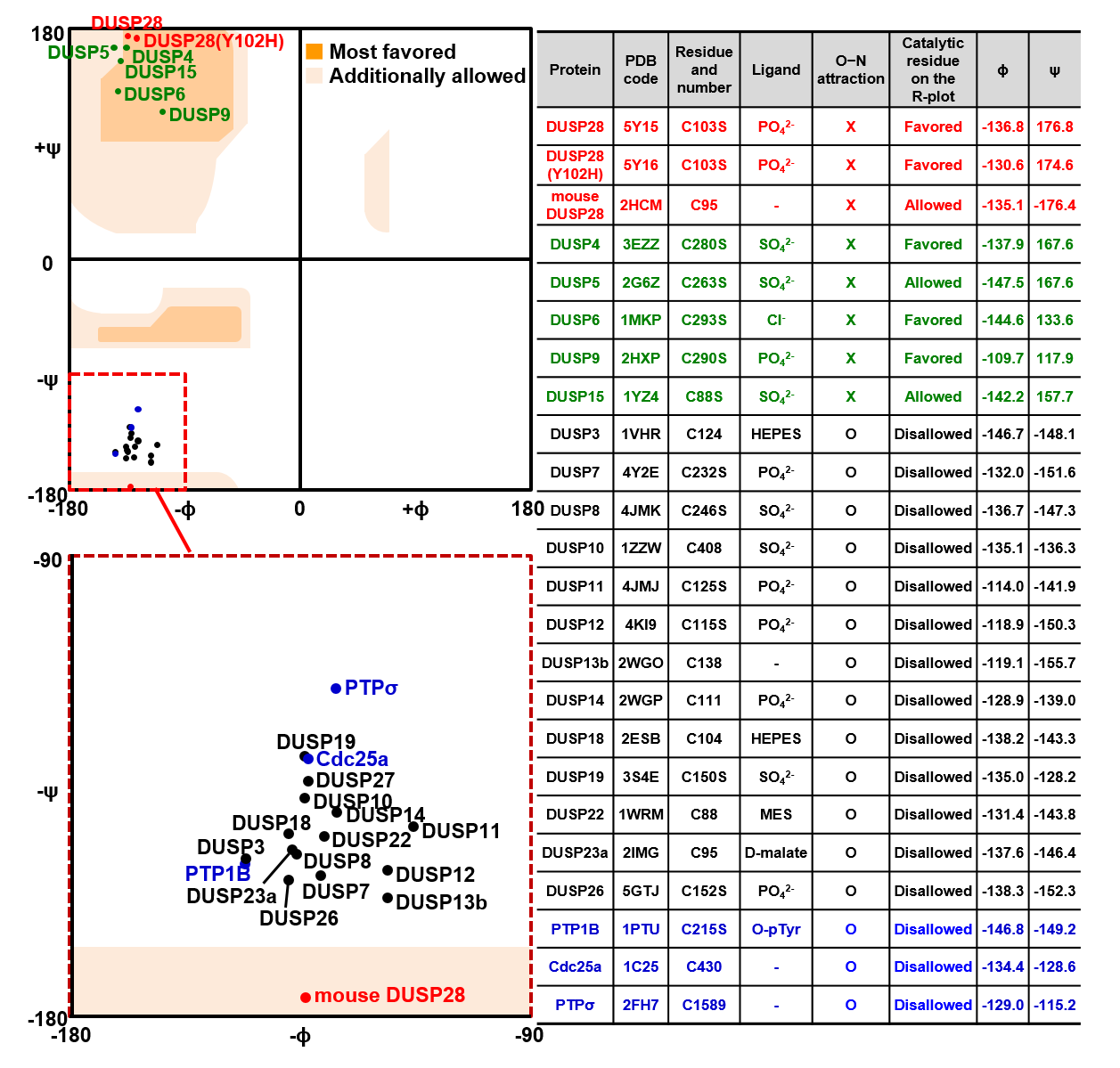

Supplement: S3 Fig — Main chain dihedral angles of the catalytic residues of 20 DUSPs and three non-DUSP PTPs (blue) are presented on the Ramachandran plot diagram. DUSP28 proteins are red, and the rest DUSPs within the favored or allowed region are green; all the other DUSP proteins are black. Detailed information about the 23 proteins is listed on the right. “O-N attraction” indicates the presence of the attraction between the histidine residue ahead of the catalytic cysteine and the main chain carbonyl group of the catalytic residue. (TIF) [file pone.0187701.s003.tif]

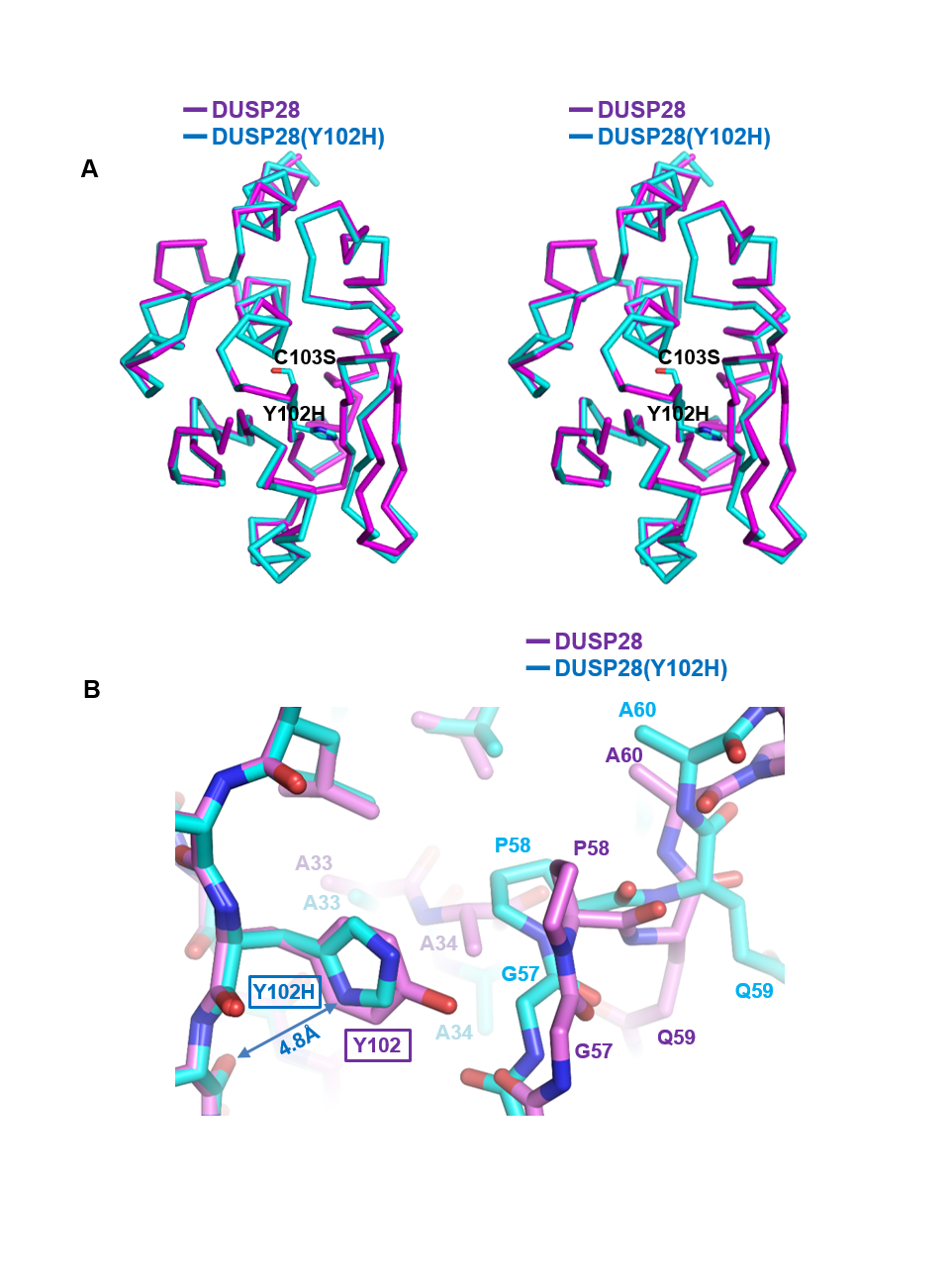

Supplement: S4 Fig — (A) Stereo views of the superimposed structures of DUSP28 in violet and DUSP28(Y102H) in cyan shown as Cα trace representation. C103S and Y102H residues of DUSP28(Y102H) are presented as sticks with labels. (B) Stick models of DUSP28 and DUSP28(Y102H) are structurally aligned and compared. The substituted residues (highlighted by rectangles) as well as residues showing conformational alteration between the two structures are labeled. Distance between the Nδ atom of the substituted histidine residue and the main chain carbonyl group of the catalytic residue is presented with an arrow; it is longer than the limit of distance for an energetically significant hydrogen bond, 3.5 Å. (TIF) [file pone.0187701.s004.tif]
